# Supplementary material for: Effect of Sarcopenia on Survival and Health-Related Quality of Life in Patients with Hepatocellular Carcinoma after Hepatectomy
Source: Cancers (Basel). 2022 Dec 13;14(24):6144. doi: 10.3390/cancers14246144 (PMC9776353; doi:10.3390/cancers14246144)
Supplement: Supplementary file 1 [file cancers-14-06144-s001.zip › Supplementary File/Table S1.docx]

Table S1. Clinical characteristics and outcomes of the 129 patients who completed the QLQ-C30 questionnaire, including those who chose open surgery or laparoscopic surgery.

| **Characteristics** | **Total population**  **N=129** | **Laparoscopy group**  **N=67** | **Open group**  **N=62** | ***p*-Value** |
| --- | --- | --- | --- | --- |
| Age, n (%)  16-60  61-82  Sex, n (%)  Female  Male  Medical insurance, n (%)  No  Yes  Annual income, yuan  0–49,999  50,000~  Preoperative ascites, n (%)  No  Yes  Cirrhosis, n (%)  No  Yes  Child cough, n (%)  A  B  TNM, n (%)  I  II-IV  Tumor size, n (%)  0.1cm-5.0cm  ≥5.1cm  ASA grade, n (%)  I-II  III  Blood loss, n (%)  ≤400 ml  >400 ml  Transfuse blood, n (%)  No  Yes  BCLC, n (%)  0-A  B-C  AFP, n (%)  ≤400 ng/ml  >400 ng/ml  Location, n (%)  Left liver  Right liver  Both sides  Lesions, n (%)  Solitary  Multiple  Surgery, n (%)  Laparoscopy  Open  Degree of differentiation, n (%)  Low  Middle  High  Satellite stove, n (%)  No  Yes  Vascular invasion, n (%)  No  Yes  Albumin, n (%)  ≥40 g/L  <40 g/L  Physical functioning  Role functioning  Emotional functioning  Cognitive functioning  Social functioning  Fatigue  Nausea and vomiting  Pain  Dyspnea  Insomnia  Appetite loss  Constipation  Diarrhea  Financial difficulties  Global health status | 65(50.4)  64(49.6)  18(14)  111(86.0)  5(3.9)  124 (96.1)  101(78.3)  28 (21.7)  9(7.0)  120(93)  47(36.4)  82(63.6)  125(96.9)  4(3.1)  82(63.6)  47(36.4)  108(83.7)  21(16.3)  116(89.9)  13 (10.1)  114(88.4)  15 (11.6)  67(51.9)  62 (48.1)  89(69.0)  40 (31.0)  111(86)  18 (14.0)  30(23.3)  89(69.0)  10(7.8)  116(89.9)  13(10.1)  67(51.9)  62(48.1)  31 (24.0)  80 (62.0)  18 (14.0)  118(91.5)  11(8.5)  119(92.2)  10 (7.8)  69(53.5)  60 (46.5)  93.80(13.54)  94.06(16.77)  83.79(16.38)  90.83(16.00)  89.53(19.88)  15.68 (19.47)  0.90(6.36)  8.01(12.52)  6.72(16.86)  19.90(31.05)  3.88(14.21)  8.53 (20.95)  3.62(15.16)  25.06(34.62)  72.09(25.76) | 30(44.8)  37(55.2)  9(13.4)  58(86.6)  2(3.0)  65(97.0)  51(76.1)  16 (23.9)  6(9.0)  61(91)  23(34.3)  44(65.7)  65(97)  2(3.0)  49(73.1)  18(26.9)  61(91)  6(9.0)  63(94.0)  4 (6.0)  63(94.0)  4 (6.0)  36(53.7)  31 (46.3)  51(76.1)  16 (23.9)  58(86.6)  9 (13.4)  20(29.9)  43(64.2)  4(6.0)  64(95.5)  3(4.5)  67(100.0)  0(0.0)  12 (17.9)  45 (67.2)  10 (14.9)  65(97)  2(3.0)  62(92.5)  5 (7.5)  36(53.7)  31 (46.3)  94.83(13.07)  95.27(14.74)  87.94(11.90)  90.80(13.70)  95.27(12.24)  11.61 (15.04)  0.25(2.04)  5.47(11.01)  2.49(8.83)  20.90(30.61)  1.99(9.85)  5.97 (17.34)  1.00(5.72)  12.94(25.26)  76.74(22.55) | 35(56.5)  27(43.5)  9(14.5)  53(85.5)  3(4.8)  59 (95.2)  50(80.6)  12 (19.4)  3(4.8)  59(95.2)  24(38.7)  38(61.3)  60(96.8)  2(3.2)  33(53.2)  29(46.8)  47(75.8)  15(24.2)  53(85.5)  9 (14.5)  51(82.3)  11 (17.7)  31(50.0)  31 (50.0)  38(61.3)  24 (38.7)  53(85.5)  9 (14.5)  10(16.1)  46(74.2)  6(9.7)  52(83.9)  10(16.1)  0(0.0)  62(100.0)  19 (30.6)  35 (56.5)  8 (2.9)  53(85.5)  9(14.5)  57(91.9)  5 (8.1)  33(53.2)  29 (46.8)  92.69(14.06)  92.74(18.75)  79.30(19.25)  90.86(18.28)  83.33(24.33)  20.07 (22.66)  1.61(8.91)  10.75(13.53)  11.29(21.71)  18.82(31.72)  5.91(17.62)  11.29 (24.10)  6.45(20.77)  38.17(38.58)  67.07(28.17) | 0.251  1.000  0.381  0.682  0.568  0.739  1.000  0.030  0.035  0.187  0.070  0.805  0.103  1.000  0.162  0.057  <0.001  0.239  0.043  1.000  1.000  0.373  0.394  0.002  0.982  0.001  0.013  0.225  0.016  0.003  0.706  0.117  0.150  0.041  <0.001  0.033 |

Abbreviations: ASA, American Society of Anesthesiologists; AFP, alpha-fetoprotein.
