# Supplementary material for: Effect of Sarcopenia on Survival and Health-Related Quality of Life in Patients with Hepatocellular Carcinoma after Hepatectomy
Source: Cancers (Basel). 2022 Dec 13;14(24):6144. doi: 10.3390/cancers14246144 (PMC9776353; doi:10.3390/cancers14246144)
Supplement: Supplementary file 1 [file cancers-14-06144-s001.zip › Supplementary File/Table S2.docx]

Table S2. Comparison of baseline information stratified by surgical approach in HCC patients with sarcopenia and without sarcopenia.

| **Characteristics** | **Patients with sarcopenia (n=34)**  **Laparoscopy group Open group *p*-Value**  **N=20 N=14** | | | **Patients without sarcopenia (n=95)**  **Laparoscopy group Open group *p*-Value**  **N=47 N=48** | | |
| --- | --- | --- | --- | --- | --- | --- |
| Age, n (%)  16-60  61-82  Sex, n (%)  Female  Male  Preoperative ascites, n (%)  No  Yes  Cirrhosis, n (%)  No  Yes  Child cough, n (%)  A  B  TNM, n (%)  I  II-IV  Tumor size, n (%)  0.1cm-5.0cm  ≥5.1cm  ASA grade, n (%)  I-II  III  Blood loss, n (%)  ≤400 ml  >400 ml  BCLC, n (%)  0-A  B-C  AFP, n (%)  ≤400 ng/ml  >400 ng/ml  Location, n (%)  Left liver  Right liver  Both sides  Lesions, n (%)  Solitary  Multiple  Surgery, n (%)  Laparoscopy  Open  Degree of differentiation, n (%)  Low  Middle  High  Satellite stove, n (%)  No  Yes  Vascular invasion, n (%)  No  Yes  Albumin, n (%)  ≥40 g/L  <40 g/L  Physical functioning  Role functioning  Emotional functioning  Cognitive functioning  Social functioning  Fatigue  Nausea and vomiting  Pain  Dyspnea  Insomnia  Appetite loss  Constipation  Diarrhea  Financial difficulties  Global health status | 7(35)  13(65.0)  2(10.0)  18(90.0)  17(85)  3(15.0)  7(35)  13(65.0)  18(90.0)  2(10.0)  15(75)  5(25.0)  19(95)  1(5.0)  19(95)  1 (5.0)  19(95)  1 (5.0)  14(70)  6 (30.0)  18(90)  2 (10.0)  4(20.0)  16(80.0)  0(0.0)  19(95)  1(5.0)  20(100.0)  0(0.0)  4 (20.0)  13 (65.0)  3 (15.0)  19(95)  1(5.0)  19(95)  1 (5.0)  9(45)  11 (55.0)  92.67(14.65)  95.00(15.39)  90.42(12.47)  93.33(11.34)  90.83(15.74)  10.56 (15.91)  0.00(0.00)  1.67(5.13)  1.67(7.45)  21.67(31.11)  1.67(7.45)  11.67 (27.09)  0.00(0.00)  15.00(22.88)  79.58(21.88) | 7(50.0)  7(50.0)  0(0.0)  14(100.0)  14(100)  0(0.0)  4(28.6)  10(71.4)  14(100.0)  0(0.0)  9(64.3)  5(35.7)  13(92.9)  1(7.1)  9(64.3)  5 (35.7)  10(71.4)  4 (28.6)  11(78.6)  3 (21.4)  12(85.7)  2 (14.3)  0(0.0)  12(85.7)  2(14.3)  9(64.3)  5(35.7)  0(0.0)  14(100.0)  6 (42.9)  8 (57.1)  0 (0.0)  13(92.9)  1(7.1)  13(92.9)  1 (7.1)  7(50)  7 (50.0)  89.52(16.89)  89.29(19.18)  79.17(16.91)  92.86(19.30)  75.00(28.31)  28.57 (23.76)  0.00(0.00)  8.33(12.66)  19.05(31.25)  19.05(38.60)  7.14(19.30)  9.52 (20.37)  19.05(31.25)  40.48(37.39)  53.57(29.73) | 0.603  0.632  0.366  0.983  0.632  0.770  1.000  0.064  0.156  0.871  1.000  0.058  0.064  <0.001  0.162  1.000  1.000  1.000  0.567  0.343  0.032  0.928  0.045  0.012  NA  0.041  0.022  0.828  0.256  0.804  0.010  0.019  0.006 | 23(48.9)  24(51.1)  7(14.9)  40(85.1)  44(93.6)  3(6.4)  16(34)  31(66.0)  47(100.0)  0(0.0)  34(72.3)  13(27.7)  42(89.4)  5(10.6)  44(93.6)  3 (6.4)  44(93.6)  3 (6.4)    37(78.7)  10 (21.3)  40(85.1)  7 (14.9)  16(34.0)  27(57.4)  4(8.5)  45(95.7)  2(4.3)  47(100.0)  0(0.0)  8 (17.0)  32 (68.1)  7 (14.9)  46(97.9)  1(2.1)  43(91.5)  4 (8.5)  27(57.4)  20 (42.6)  95.74(12.39)  95.39(14.62)  86.88(11.62)  89.72(14.56)  97.16(10.02)  12.06(14.80)  0.35(2.43)  7.09(12.41)  2.84(9.40)  20.57(30.73)  2.13(10.78)  3.55 (10.39)  1.42(6.80)  12.06(26.40)  75.53(22.95) | 28(58.3)  20(41.7)  9(18.8)  39(81.2)  45(93.8)  3(6.2)  20(41.7)  28(58.3)  46(95.8)  2(4.2)  24(50.0)  24(50.0)  34(70.8)  14(29.2)  44(91.7)  4 (8.3)  41(85.4)  7 (14.6)  27(56.2)  21 (43.8)  41(85.4)  7 (14.6)  10(20.8)  34(70.8)  4(8.3)  43(89.6)  5(10.4)  0(0.0)  48(100.0)  13 (27.1)  27 (56.2)  8 (16.7)  40(83.3)  8(16.7)  44(91.7)  4 (8.3)  26(54.2)  22 (45.8)  93.61(13.19)  93.75(18.71)  79.34(20.05)  90.28(18.14)  85.76(22.80)  17.59(21.96)  2.08(10.10)  11.46(13.82)  9.03(17.85)  18.75(29.90)  5.56(17.30)  11.81 (25.25)  2.78(15.12)  37.50(39.28)  71.01(26.74) | 0.476  0.820  1.000  0.579  0.484  0.043  0.045  1.000  0.333  0.922  1.000  0.337  0.449  <0.001  0.434  0.039  1.000  0.908  0.419  0.636  0.028  0.868  0.002  0.154  0.257  0.109  0.038  0.771  0.251  0.041  0.575  <0.001  0.379 |

Abbreviations: ASA, American Society of Anesthesiologists; AFP, alpha-fetoprotein.
