# Supplementary material for: Effect of Sarcopenia on Survival and Health-Related Quality of Life in Patients with Hepatocellular Carcinoma after Hepatectomy
Source: Cancers (Basel). 2022 Dec 13;14(24):6144. doi: 10.3390/cancers14246144 (PMC9776353; doi:10.3390/cancers14246144)
Supplement: Supplementary file 1 [file cancers-14-06144-s001.zip › Supplementary File/Table S3.docx]

Table S3. Linear regression analysis of surgical approach on health-related quality of life after adjusting for confounders separately in the total population, patients with sarcopenia and patients without sarcopenia.

| **Characteristics** |  | **Total population** | |  | **Patients with sarcopenia** | |  | **Patients without sarcopenia** | |
| --- | --- | --- | --- | --- | --- | --- | --- | --- | --- |
|  | | **β (95%CI) *p*-value** | |  | **β (95%CI)** | ***p*-value** | | **β (95%CI)** | ***p*-value** |
| Physical functioning  Role functioning  Emotional functioning  Cognitive functioning  Social functioning  Fatigue  Nausea and vomiting  Pain  Dyspnea  Insomnia  Appetite loss  Constipation  Diarrhea  Financial difficulties  Global health status | | -1.46 (-6.23, 3.31)  -2.47 (-8.52, 3.59)  -9.58 (-15.38, -3.79)  -1.81 (-7.61, 3.99)  -10.68 (-17.60, -3.75)  9.00 (2.18, 15.83)  0.75 (-1.45, 2.96)  4.08 (-0.34, 8.49)  7.50 (1.56, 13.43)  0.15 (-11.27, 11.57)  3.30 (-1.70, 8.31)  5.21 (-2.23, 12.65)  6.06 (0.54, 11.57)  21.87 (10.18, 33.55)  -8.02 (-17.29, 1.26) | 0.545  0.421  0.001  0.539  0.003  0.010  0.501  0.070  0.014  0.979  0.194  0.168 0.032  <0.001  0.090 | | -3.14 (-14.22, 7.93)  -5.71 (-17.80, 6.37)  -11.25 (-21.50, -1.00)  -0.48 (-11.19, 10.23)  -15.83 (-31.26, -0.40)  18.02 (4.19, 31.84)  -0.00 (0.00, 0.00)  6.67 (0.29, 13.04)  17.38 (2.67, 32.10)  -2.62 (-27.00, 21.76)  5.48 (-4.16, 15.11)  -2.14 (-19.59, 15.31)  19.05 (4.91, 33.19)  25.48 (4.44, 46.52)  -26.01 (-44.01, -8.01) | 0.567  0.343  0.032  0.928  0.045  0.012  NA  0.041  0.022  0.828  0.256  0.804  0.010  0.019  0.006 | | -1.33 (-6.69, 4.04)  -1.58 (-8.82, 5.66)  -9.40 (-16.50, -2.30)  -1.79 (-8.80, 5.23)  -8.76 (-16.28, -1.24)  6.08 (-1.95, 14.11)  0.84 (-2.16, 3.84)  3.13 (-2.41, 8.67)  4.23 (-1.91, 10.36)  0.14 (-13.08, 13.36)  2.48 (-3.53, 8.49)  7.82 (-0.11, 15.76)  2.17 (-2.97, 7.31)  20.39 (6.13, 34.66)  -2.25 (-13.07, 8.57) | 0.625  0.666  0.010  0.614  0.023  0.136  0.579  0.265  0.174  0.984  0.415  0.053  0.405  0.006  0.680 |
