# Supplementary figures and images for: Effect of Sarcopenia on Survival and Health-Related Quality of Life in Patients with Hepatocellular Carcinoma after Hepatectomy
Source: Cancers (Basel). 2022 Dec 13;14(24):6144. doi: 10.3390/cancers14246144 (PMC9776353; doi:10.3390/cancers14246144)

a

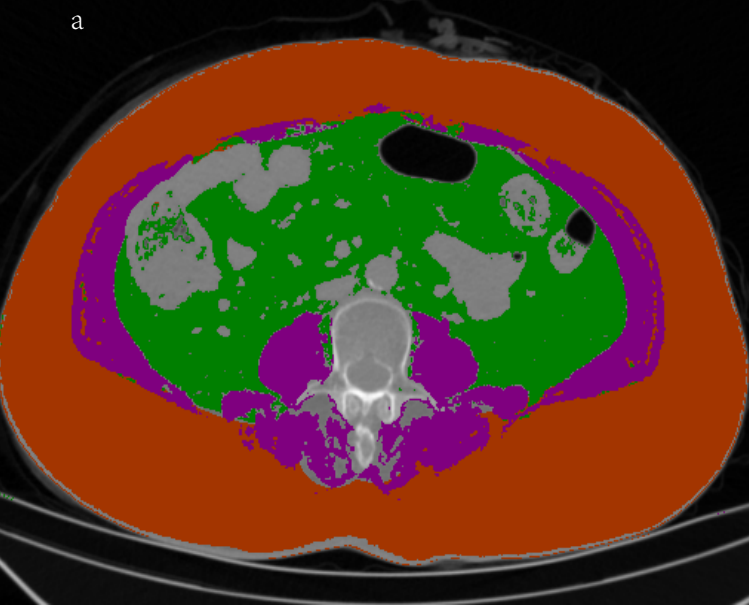

b

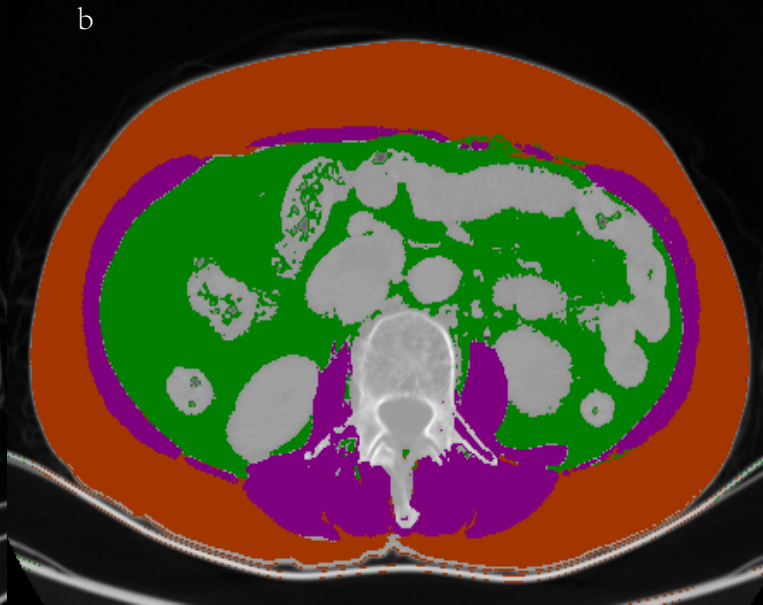

c

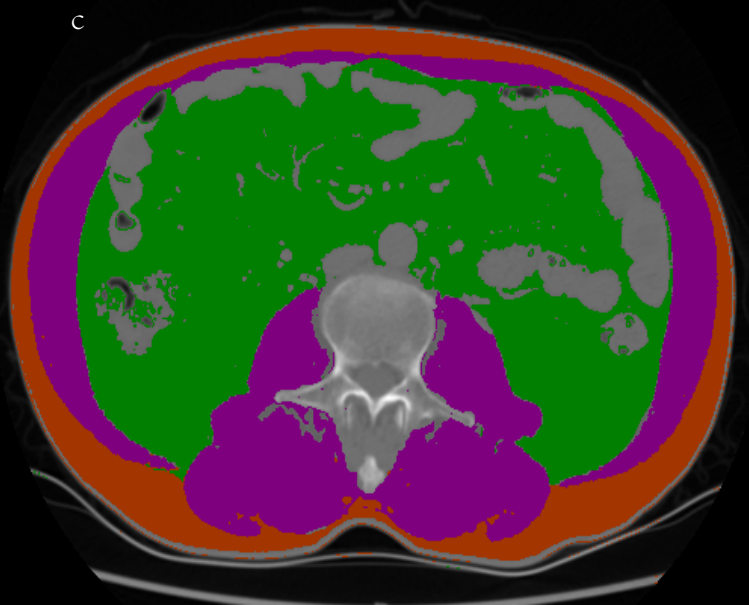

d

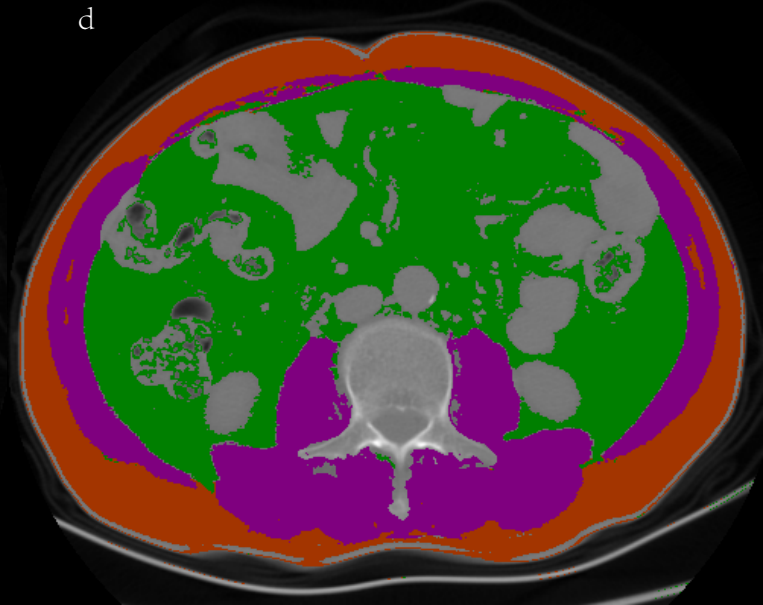

Supplement: Supplementary file 1 [file cancers-14-06144-s001.zip › Supplementary File/Figure S1.pdf]
